# Brain Computer Interfaces for Improving the Quality of Life of Older Adults and Elderly Patients

**DOI:** 10.3389/fnins.2020.00692

**Published:** 2020-06-30

**Authors:** Abdelkader Nasreddine Belkacem, Nuraini Jamil, Jason A. Palmer, Sofia Ouhbi, Chao Chen

**Affiliations:** ^1^Department of Computer and Network Engineering, College of Information Technology, United Arab Emirates University, Al Ain, United Arab Emirates; ^2^Department of Computer Science and Software Engineering, College of Information Technology, United Arab Emirates University, Al Ain, United Arab Emirates; ^3^Department of Neurological Diagnosis and Restoration, Osaka University, Suita, Japan; ^4^Key Laboratory of Complex System Control Theory and Application, Tianjin University of Technology, Tianjin, China

**Keywords:** brain computer interface, EEG, cognitive aging, motor impairment, older adults, elderly patients

## Abstract

All people experience aging, and the related physical and health changes, including changes in memory and brain function. These changes may become debilitating leading to an increase in dependence as people get older. Many external aids and tools have been developed to allow older adults and elderly patients to continue to live normal and comfortable lives. This mini-review describes some of the recent studies on cognitive decline and motor control impairment with the goal of advancing non-invasive brain computer interface (BCI) technologies to improve health and wellness of older adults and elderly patients. First, we describe the state of the art in cognitive prosthetics for psychiatric diseases. Then, we describe the state of the art of possible assistive BCI applications for controlling an exoskeleton, a wheelchair and smart home for elderly people with motor control impairments. The basic age-related brain and body changes, the effects of age on cognitive and motor abilities, and several BCI paradigms with typical tasks and outcomes are thoroughly described. We also discuss likely future trends and technologies to assist healthy older adults and elderly patients using innovative BCI applications with minimal technical oversight.

## Introduction

Aging has its effects on human body and brain, especially in the molecules, cells, vasculature, gross morphology, and cognition. This biological aging becomes disability and dependence as time goes by. Many researchers have been suggesting transdisciplinary approaches to address the problem of aging and its effects on activities of daily living. Healthy older adults and elderly patients may have difficulties in communicating, concentrating, memorizing, talking, walking, or maintaining balance. These deficits may lead to inability to communicate with their family, climb stairs, memorize new information, or drive safely. The aging process does not affect people uniformly, but most elderly need to use assistive technologies to better perform daily-life activities if only the need for handrails or canes to get up steps. Unfortunately, they do not receive the support they need, because the cost of caring for them runs into the billions of dollars. Brain computer interface (BCI) technology is now being incorporated into the treatment of many patients suffering from cognitive or physical impairments. This technology offers the promise of greatly enhancing these patients’ quality of life by considerably improving their personal autonomy and mobility. BCI can be used as an assistive, adaptive, and rehabilitative technology to monitor the brain activity and translate specific signal features that reflect the elderly’s intent into commands that operate any device. BCI systems could be useful for elderly people in many ways such as: (1) training their motor/cognitive abilities for preventing the aging effects, (2) controlling home appliances, (3) communicating with others during daily activities, and (4) controlling an exoskeleton to enhance the strength of the body’s joints. The purpose of this mini-review is to survey some examples where BCIs can be feasible and useful medical and non-medical applications for healthy older adults and elderly patients using non-invasive measurements such as electroencephalogram (EEG) to improve their quality of life.

The topic is introduced further in the following subsections. In subsection “Age-related Changes,” age-related brain and body changes are reviewed. In subsection “Brain computer interface technology,” the principle and paradigm of several types of BCI are reviewed. In subsection “Aging and cognitive abilities,” the impact of aging on cognitive abilities is discussed. Finally, common health conditions associated with aging such as motor control impairments are given in subsection “Aging and motor control impairments.”

### Age-Related Changes

Human development encompasses multiple phases, including baby, toddler, teenager, adult, and old age. Throughout the aging process, some of the most profound changes involve brain cognition. Cognition is an essential aspect of human information processing. According to social development perspectives, a person’s brain will begin to decline gradually as the individual reaches middle adulthood and will continue to decline throughout the aging process (Peters, 2006). Recalling memories and learning new skills become more difficult and can take a longer time. Both declarative memory and procedural memory can be affected. Life routines stored in declarative memory will slowly change and be forgotten due to aging. This can be most pronounced in Alzheimer’s disease. Deterioration in procedural memory can make it very difficult to learn new skills such as a new language (Quam et al., 2018). Lifestyle changes as a result of the aging process will often affect both elderly persons and their family members. Healthy elderly people often report a decline in memory that causes them to experience depression and anxiety (Hertzog et al., 2000). As people age, they may also have difficulty paying attention to multiple tasks. For example, at a traffic light, the processing of the information about the light changing can distract from processing of other surroundings, and thus can lead to road accidents.

Additionally, the brain parenchyma will shrink and change along with the adjustment in cognitive ability. Brain shrinkage appears especially significant in medial temporal lobe structures and tertiary association cortices (i.e., regions that are particularly important for support of age-sensitive cognitive functions). In contrast, sensory cortical regions (i.e., the visual cortex) evidence lesser age-related change (Persson et al., 2016). This shrinking will affect memory and mental sharpness. When a person gets older, the brain shrinks naturally. Changes in the brain structure can reduce the communication between neurons in some parts of the brain. Blood flow to the brain will also decrease. Changes may also take place in the neurotransmitter system, which can cause depression and other mood disorders (Nutt, 2008).

As humans age, the risk factors for brain diseases such as Alzheimer’s, dementia, heart attack, depression, and obesity increase. All of these diseases can contribute to damage to brain structure and decreased brain function (Uylings and De Brabander, 2002), leading to reduced cognitive and memory function. These progressions can influence the capacity to encode new data into memory and retrieve data already in memory. Healthy lifestyle, including physical, and mental exercise, might be one of the ways to prevent changes.

Aging affects more than just the brain. Muscles commonly start to lose the function and become slow and weak (sarcopenia; Ryall et al., 2008). Strength gradually decreases (frailty) and contributes toward the limitation of physical activity such as running, hiking and general social wellbeing (Fried et al., 2001). Furthermore, neurodegenerative diseases as Parkinson’s become a risk for people over age 60. The motor system affected by the degenerative impairment of the central nervous system can cause limb tremor, postural instability and stiffness (Willis, 2013). Physical conditioning and the use of advanced technologies such as BCI to assist mobility and performance of fine motor control can greatly improve the quality of life for the elderly.

### Brain Computer Interface Technology

Brain computer interface is one of the most promising and increasingly popular technologies for assisting and improving communication/control for motor paralysis (e.g., paraplegia or quadriplegia) due to stroke, spinal cord injury, cerebral palsy, and amyotrophic lateral sclerosis (ALS). Eye-tracking technology also allows paralyzed people to control external devices but it has many drawbacks due to the way of measuring the eye movements via cameras or using attached electrode on face such as electrooculography (EOG) signals. BCI essentially involves translating human brain activity into external action by sending neural commands to external devices (Belkacem et al., 2015a, 2018; Gao et al., 2017; Chen et al., 2020; Shao et al., 2020). Although, the most common use of BCI is to help disabled people with disorders in the motor system, it might be very useful tool for improving the quality of life of healthy people, particularly the elderly. Assistive, adaptive, and rehabilitative BCI applications for older adults and elderly patients should be developed to assist with their domestic chores, enhance relationships with their families and improve their cognitive and motor abilities. BCI technology has clinical and non-clinical applications in many areas, including medicine, entertainment, education, and psychology to solve many health issues such as cognitive deficits, slowness in processing speed, impaired memory and movement capability decline among elderly people. These issues can affect the quality of elderly life and may have adverse effects on mental health. To help older people maintain a healthy, good quality of life and sense of wellbeing, many BCI applications have been developed in the past decade.

There are two types of BCI based on the electrodes used for measuring the brain activity: non-invasive BCI where the electrodes are placed on the scalp (e.g., EEG based BCI), and invasive Brain computer interface where the electrodes are directly attached on human brain [e.g., BCI based on electrocorticography (ECoG), or intracranial electroencephalography (iEEG)].

Brain computer interfaces using EEG technology have been widely used to establish portable synchronous and asynchronous control and communication. Non-invasive EEG-based BCIs can be classified as “evoked” or “spontaneous.” An evoked BCI exploits a strong characteristic of the EEG, the so-called evoked potential, which reflects the immediate automatic responses of the brain to some external stimuli. Spontaneous BCIs are based on the analysis of EEG phenomena associated with various aspects of brain function related to mental tasks carried out by the BCI user at their own will. These BCIs have been developed based on some brain features such as evoked potentials [e.g., P300 and steady-state visual evoked potential (SSVEP)] or based on slow potential shifts and variations of rhythmic activity [e.g., motor imagery (MI)].

To build a BCI system, five or six components are generally needed: signal acquisition during a specific experimental paradigm, preprocessing, feature extraction (e.g., P300 amplitude, SSVEP, or alpha/beta bands), classification (detection), translation of the classification result to commands (BCI applications), and user feedback. For quick and accurate processing and analysis of brain data, researchers have developed many open source software packages and toolboxes such as BCI2000^1^, EEGLab^2^, FieldTrip^3^, and Brainstorm^4^. These software packages are based on advanced signal and image processing methods and artificial intelligence programs for performing sensor or source level analyses (Belkacem et al., 2015b, 2020; Dong et al., 2017).

However, many critical issues are faced in the development of a ready-to-use BCI product. These critical issues include low classification accuracy, small number of degrees of freedom, and long training time to learn how to perfectly operate a BCI. Therefore, researchers have been trying to improve the performance of the existing BCIs by developing a hybrid BCI (hBCI) that combines at least two BCI modalities (e.g., P300 with SSVEP or P300 with MI). The hBCI combines different approaches to utilize the advantages of multiple BCI modalities. It can be also a combination of brain activity with non-brain activity, and various other psychological signals were shown to be a promising option of hBCI development (Scherer et al., 2007; Choi et al., 2016). Thus, the input signals can consist of the combination of two brain characteristics using EEG signals, or EEG with eye movements (EOG), muscle activity (electromyography, EMG), or with heart signal (ECG or EKG). However, P300-based BCIs (e.g., a visual/auditory/tactile P300 Speller) are the most popular BCI systems due to their high classification accuracy and speed, or information transfer rate (ITR).

In addition, a closed-loop BCI system using visual and proprioceptive feedback with real-time modulation and communication can be used not only for interacting with the external environment, but also as a biofeedback platform to enhance the cognitive abilities of elderly patients and provide better therapeutic effects. This closed-loop interaction between the participant’s brain responses and the stimuli is thought to induce cerebral plasticity and thereby facilitate rehabilitation.

One of the greatest challenges in BCI technology is the development of less invasive or non-invasive technologies for paralyzed patients. Using non-invasive devices can greatly reduce the both the total cost of surgical operation and the physical harm to the patient. However, non-invasive methods can lead to weaker signals and a low signal-to-noise ratio (SNR) with less source precision and lower spatial resolution. These drawbacks can be partially overcome with advanced methods such as deep learning to decode and extract more relevant source information from the EEG signal (Nagel and Spüler, 2019).

Electroencephalogram -based BCI technology has many important applications in the medical and psychology fields not only for motor control impairments. One promising application for elderly patients is the development of automatic systems to detect the influences on the brain signal related the smoking and alcohol abuse using resting-state EEG (Mumtaz et al., 2017; Su et al., 2017). BCI has also been found to be helpful in identifying deficits and improving social skills in patients with autism through the use of BCI-assisted social games (Amaral et al., 2018). Other research has focused on systems to test memory capacity and cognitive level (Burke et al., 2015; Buch et al., 2018).

### Aging and Cognitive Abilities

Old age is a key risk factor for many major medical health problems, not least neurodegenerative disease and dementia. In fact, a number of neurological and psychiatric diseases (e.g., schizophrenia, depression, epilepsy, HIV infection, and traumatic brain injury) have been proposed to result in premature or accelerated aging, based on clinical observations and behavioral or biological research (Cole et al., 2019). Invasive techniques (e.g., deep brain stimulation) and non-invasive measurements [e.g., EEG and functional magnetic resonance imaging (fMRI)] have been used to treat and/or understand the pathophysiology of schizophrenia, depression, and epilepsy using specified regions of interest (ROIs), quantitative EEG (brain mapping), or EEG rhythms (e.g., delta, theta, alpha, beta, and gamma bands). However, one of the most common aging-related health issues after cardiovascular conditions is dementia, which may be caused by diseases such as Alzheimer’s, Lewy body disease, vascular dementia and frontotemporal dementia. Patients with dementia may lose the ability to think clearly, learn and remember. We focus in the following paragraphs on memory impairments and how BCI technology can prevent, reduce, or solve them.

Memory is stored in the human brain to keep information and previous experiences available for recall whenever needed. Memory helps people to learn from past experiences, and assists in acquiring new skills and learning new information. From a neurological and psychological perspective, human memory involves grouping and communication between neurons in the human brain. Human memory is not located in only one area of the human brain, but rather involves the cooperation of several areas. Memory and learning are strongly related in term sharing almost the same brain areas, but in term of brain mechanism and process are strictly distinct from one and another. Memory itself is consecutive from retrieving the knowledge and adjust with our behavior but learning is the process when the neurons are working up to gain the knowledge and information.

In general, human memory is divided into short-term memory and long-term memory. Short-term memory, also known as working memory, is temporary storage that can hold a smaller amount of memory that can be accessed immediately. For example, remembering a phone number that was just mentioned, or a secure number from the bank for the transaction involves short-term memory. Information can be retrieved after only a few seconds in our short-term memory. In contrast, long-term memory lasts over an extended period of time, and can store a much larger amount of information (Konkle et al., 2010). Working memory can be transferred to long-term memory through rehearsal and strengthening.

Human memory can start to degrade from age 20. Memory loss can be one of the worst factors associated with the aging process. The risk of developing memory-related diseases like dementia and Alzheimer’s proportionally increase with age. Older people tend to have difficulty remembering or recognizing objects in the same group and semantic category (Pansuwan et al., 2020). For example, different types of animal-like hamsters or dogs in the same class can cause confusion and incorrect recognition. As the brain ages, some regions become slower due to decreased blood flow. Additionally, neurotransmitters are also reduced and affect the ability to understand the environment and access memory.

Brain computer interface technology could be one potential tool for restoring learning and improving memory, attention, and consciousness for cognitively impaired elderly patients (Buch et al., 2018). For instance, non-invasive BCIs have been be used for restoring memory and planning using electromagnetic stimulation and biofeedback that modulate activity in a patient’s brain as part of a rehabilitation program. In addition, BCIs have been used to enhance episodic memory in human participants where neural oscillations in the theta and alpha bands were used to predict the future success of memory encoding. Electrophysiological signals may also be causally linked to a specific behavioral condition, and contingent stimulus presentation has the potential to modulate human memory encoding (Burke et al., 2015). Moreover, BCI could provide a powerful approach for future applications in cognitive prosthetics (e.g., promises to improve learning and memory for patients with cognitive impairment, which need a deep understanding of the neural mechanisms underlying these cognitive processes).

### Aging and Motor Control Impairments

Motor control is a complex system that includes the brain, muscle and limb (Rosenbaum, 2009). Cooperation between physical and physiological systems makes it possible for the human body to move. Physical movements include walking, running, grabbing or exercising. Physiological control mechanisms include cholesterol levels, blood pressure and equilibrium. All of these can be destroyed due to aging factors, accidents, or disease and they typically do not heal naturally.

**FIGURE 1:**
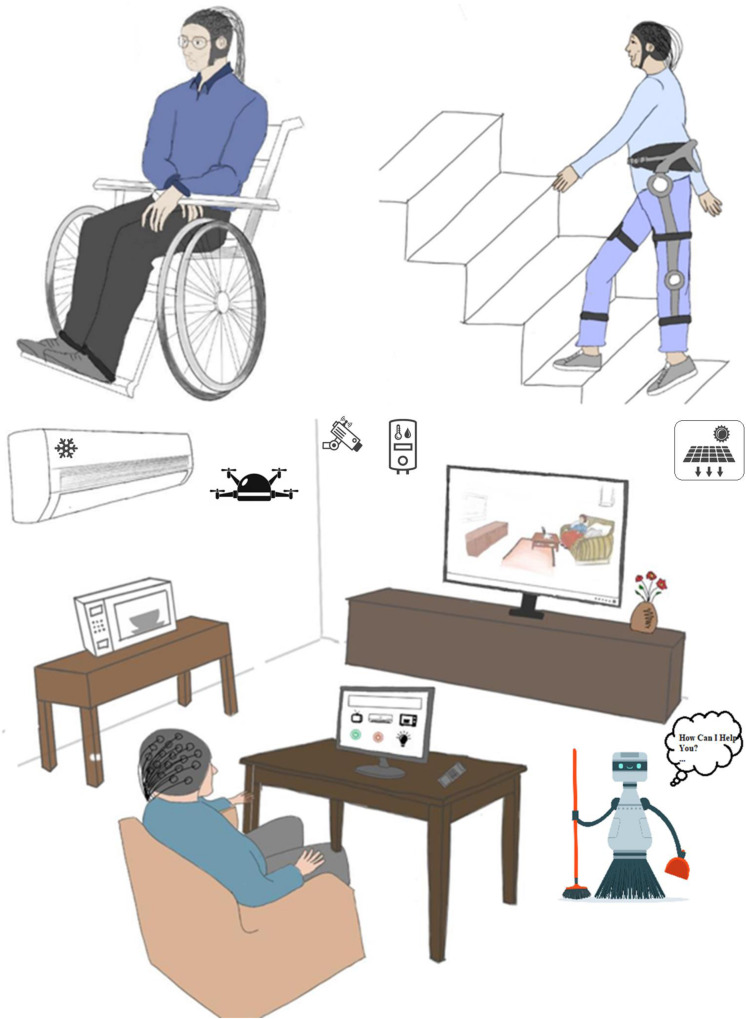
Possible assistive applications of EEG-based BCI for decreasing debilitating and dependence of elderly (e.g., controlling a wheelchair, exoskeleton “soft Exosuits,” drone, assistive robot, and smart home appliances).

Aging tends to naturally reduce motor skills and physiological energy levels. Hence, it can reduce the speed of human movements such as walking (Wert et al., 2010). The elderly can exercise and practice to improve muscle and motor skills (Kleim, 2011), however, excessive training and practice can be risky for older people and might contribute to other injury or disease. Emerging technologies using BCI can contribute to keeping healthy elderly fit. Elderly people who need assistance or rehabilitation can continue their ordinary life routines using a BCI system (see Figure 1). In the following, we note three possible EEG-based BCIs for age-related motor control impairments: controlling an exoskeleton, wheelchair, and smart home appliances (including drone and smart cleaning and/or assistive robots to perform physical tasks for the well-being of the elderly).

Robotic exoskeletons have been developed to increase joint strength and to reduce the effect of carrying a heavy load. An exoskeleton can enable a soldier to lift a heavy object, or assist a firefighter who has to wear heavy equipment. At the same time, exoskeletons can be accessories to assist elderly people or people with motor impairments in performing their daily activities. There are various types of exoskeletons for use by elderly people, such as lower limb Exosuits (Shore et al., 2020), ankle-foot exoskeleton to assist in plantarflexion while walking (Galle et al., 2017), robotic exoskeleton to facilitate the movement of shoulder and elbow (Tang et al., 2019), and upper limb exoskeleton for hand grasping and motion (Chauhan et al., 2019).

A wheelchair is a very common device used by healthy and disabled elderly to move from one place to another without external aid. The need for a wheelchair can be caused by loss of muscle strength, or diseases like ALS, arthritis or Parkinson’s. Often patients require a caretaker to help them move and perform their daily routine. However, sometimes a caretaker is not available. In this case, some extended functions are available for wheelchair use by elderly people. The automated wheelchair is one easy way for the elderly to navigate in the home (Brandt et al., 2004). The elderly can also move from one room to another room by wheelchair with voice control in combination with the navigation assistance provided by “Smart wheelchairs,” which use sensors to identify and avoid obstacles in the wheelchair’s path (Megalingam et al., 2011). Finally, an intelligent wheelchair such as RoboChair, with a head gesture-based interface, can be used for mobility with little effort (Gray et al., 2007).

On the other hand, the home can be a dangerous place, especially for older people who live alone and have health problems, as they may be prone to falling or other accidents. Smart home technologies are thus important solutions to enable seniors to live more safely in their own homes. The use of intelligent homes by the elderly increases their independence and improves their health (Sapci and Sapci, 2019). For example, eHomeSeniors focuses on the objective of detecting the elderly who fall in the home (Riquelme et al., 2019). Also, Kern et al. (2019) developed My Little Smart Personal Assistant for elderly people to interact with a vocal assistant that provides the medical services. Finally, Shang et al. (2019) designed a system to identify and observe behavior to support home care for elderly people who live alone in a house.

## Methods

In this mini-review, the authors conducted a literature search of available sources describing issues relating to elderly with BCI, EEG, cognitive aging, and motor control impairments. Recent research studies were selected based on research topics found in globally acknowledged databases such as Web of Science, PubMed, Springer, IEEEXplore, and Scopus. Searches were restricted to recent original ideas published inn reputable journals in the past 10 years. Papers that were not English, gray literature, book chapters, conference proceedings and opinions pieces were not included. The inclusion criteria were applied for the BCI applications that can help the aging people by reviewing titles and abstracts based on keywords. We excluded many research papers for many reasons such as redundancy, title and abstract are unrelated to the research topic, or if we could not find any occurrence of at least elderly (or older adults, elderly patients, etc.) with one keyword (BCI, EEG, cognitive aging, motor impairment, exoskeleton, wheelchair, smart home control, free communication, disorders of consciousness, rehabilitation, and neurofeedback, etc.). Due the limited available BCI articles that used the elderly people as subjects or participants, some potential BCI study designs were considered for inclusion. Thus, some studies were included with non-elderly participants if their outcomes seem to be helpful and useful for healthier elderly living. The selected articles were classified according to their relevancy (see Table 1). The information provided in the selected recent studies (e.g., age of the participants, invasive or non-invasive measurements, experimental paradigm, the purpose of the original study, the impact of the paper’s outcomes on elderly living such as engineering applications, and scientific findings) were carefully evaluated and discussed in the following section.

**TABLE 1:** Some interesting examples of related work for BCI applications for elderly people.

Author	Participant	BCI paradigm	Target	Results
**Aging and cognitive impairments**				
Lee et al., 2013	31 healthy elderly (aged between 60–70 years old)	Using EEG for study the cognitive abilities: memory and attention	To improve the memory and attention of elderly patients with playing the card pairing memory game. The participants need to focus their attention for open and close the card on the screen	Significant improvement in immediate memory (*p* = 0.038) visuospatial/constructional (*p* = 0.014), attention (*p* = 0.039), and delayed memory (*p* < 0.001) scores
Gomez-Pilar et al., 2016	63 subjects (more than 60 years old)	8 EEG electrodes for study the memory ability	Combines hand motor imagery tasks with memory exercise. The participants need to remember the repeated item	significant improvements (*p* < 0.01) in four cognitive functions after performing five (Neurofeedback Training sessions: visuospatial, oral language, memory, and intellectual
Foong et al., 2019	11 stroke patients (mean age 55.2 ± 11.0 years)	EEG (Motor Imagery) for study the assessment of the efficacy of EEG-based MI-BCI with visual feedback and EEG correlates of mental fatigue for upper-limb stroke rehabilitation	The participants need to imagine to move the upper arm (stroke-affected side) to reach the target in front of them	Have significant improvement from baseline until week 6 and 24 based on Fugl-meyer motor assessment (FMA). Also have significant positive correlations between frontal and central brain regions
Renton et al., 2019	17 naïve participants	EEG (SSVEP) for free communication between naïve human participants	The participants were facing two experiments: (i) to test either participant can maintain the rapid typing for free words, (ii) two participants will have free communication based on social BCI communication interface	Based on the naïve participants, the free communication is possible but the information transfer was reduced because of the textual correction during the free communication
Xiao et al., 2018	5 healthy participants (29 ± 5 years old) 15 patients of disorders of consciousness (13–73 years old)	EEG to study visual fixation assessment in patients with disorders of consciousness	Five healthy participants validated the proposed system. The DOC patients going thru two different visual fixation experiments; (i) Coma recovery scale-revised (CRS-R) based behavioral assessment, (ii) BCI based assessment	Three from the 15 patients showed visual fixation based on two experiments while one patient achieved significant online accuracy in BCI assessment. Thus, the BCI can be promising tools for assisting behavioral in CRS-R.
**Aging and motor control impairments**				
Herweg et al., 2016	10 healthy elderly (50–73 years old)	EEG (ERP) for controlling a virtual wheelchair	The participants need to reach three checkpoints using a virtual wheelchair with 14 movements commands	Average accuracy during the navigation tasks was above 90% and the optional task has an average of accuracy above 95%
Kaufmann et al., 2014	17 healthy participants (18–27 years old)	EEG (ERP) for controlling a wheelchair	The people with severe disabilities example neurodegenerative disease to control the wheelchair	11 participants successfully complete the task of reaching four checkpoints in the building
Villa-Parra et al., 2015	4 healthy subjects	EEG and surface EMG (sEMG) for study motor control impairment to control a robotic knee exoskeleton	To improve mobility and security for gait rehabilitation. The activities are focusing on stand-up/sit-down and knee flexion/extension	A combination of EEG/sEMG can be used to identify the control strategy to develop a system to help and restore users with muscular disabilities.
Lee et al., 2017	5 subjects (20–35 years old)	16 EEG electrodes for controlling an exoskeleton	The participants need to control three different directions: walk in front, turn left and turn right	All five subjects successfully complete the 3 ways navigation tasks and time decrease 10.2% from overall tasks from baseline protocol
Jafri et al., 2019	60 subjects (young < 50 and older than > 50)	EEG for controlling smart home and medical system	To design and evaluate the system for disabled and older adults to perform the daily task such as operating and control the home and medical appliances (a light bulb, a fan and digital blood pressure monitor)	The younger male reached attention level up to 74.78 with 26.2 s quicker than younger females and older people. The wireless BCI (WBCI) suitable for all people with brain and eyes are functional or the body of paralyzed
Chai et al., 2020	5 healthy people (mean age 23.8 ± 1.1 years) 5 paralyzed patients (mean age 48.8 ± 7.9 years)	EEG (SSVEP) with EMG to develop hBCI for smart home control	The participants need to control a wheelchair, nursing bed, curtain/light, television and telephones	The average accuracy is 97.5% for healthy participants and 83.6% for paralysis patients

## How Can BCI Applications Improve the Quality of Elderly Living?

With the rapidly increasing population of elderly people (Coimbra et al., 2010), there has been much interest in research involving the use of BCIs to improve, repair or enhance lost cognitive or motor function. Table 1 shows selected studies that represent different uses of BCI to improve the quality of life of the elderly, including improved cognitive function, especially memory, control of smart homes, and limb support for movement. We present the participants, the BCI paradigms that were implemented in the papers, the target or task that the participants were instructed to complete, and the result from the experiments.

To address aging-related cognitive impairments, Lee et al. (2013), and Gomez-Pilar et al. (2016) study the cognitive capabilities of elderly people related to memory. Both of the studies show that BCI and cognitive tests can improve memory ability among the elderly. In the Lee et al. (2013) experiment, participants have to play a card matching game to test their memory ability. At the same time, they need to focus on giving a command to close and open the card. In the Gomez-Pilar et al. (2016) experiment, participants have five tasks: (i) learning to imagine the hand movement, (ii) moving the cursor on the screen, (iii) moving the cursor toward the correct target, (iv) avoiding an obstacle for person walking in the screen, and (v) identifying the image from a previously displayed group that matches a newly displayed image.

Furthermore, delirium and confusional states are common mental disorders, which can lead to a disorder of consciousness (DOC) among older adults involving lack of environmental awareness. EEG-based BCI paradigms have many advantages in this problem domain. Xiao et al. (2018) developed a BCI system to assist the visual fixation of elderly patients with DOC to evaluate the visual part of the coma recovery scale-revised (CRS-R). Pan et al. (2018) used BCI to detect the emotion for DOC since they are unable to afford the motor responded to display the feelings. Some of the elderly face difficulty in communicating their needs. Hence, the free communication can be a tool for them to support the conversation (Renton et al., 2019). As age is the most significant risk factor in stroke, rehabilitation can help for restoring the ability for the motor functions. Prediction and monitoring of specific biomarkers of the motor function are being investigated to personalize the rehabilitation program (Mane et al., 2019). Stroke is also associated with mental fatigue, and memory issues. Foong et al. (2019) studied the correlations of mental fatigue during the BCI while performing the upper limb stroke rehabilitation.

Regarding aging-related motor control impairments, BCI-assisted wheelchair technology is one of the promising developments for rehabilitation and the elderly with muscle and severe motor disabilities. Herweg et al. (2016) have shown with ten healthy elderly participants the ability to train the user to control the wheelchair using EEG and the tactile event-related potential (ERP). Each participant did five sessions with a maximum of three sessions per week. The training task involved control of a virtual wheelchair using 14 commands in virtual environments. 90% accuracy was achieved for the navigation tasks. For the optional or bonus tasks, the efficiency was more than 95%. Kaufmann et al. (2014) also proposed a BCI system using EEG and ERP to control a wheelchair. There, the participants needed to navigate the wheelchair to four different checkpoints inside the building. The primary target users are people with neurodegenerative disease.

The wearable knee exoskeleton has been proposed and tested by Villa-Parra et al. (2015) with four main healthy subjects using EEG and EMG signals. The primary purpose of the device is to improve and rehabilitate the gait, and restore the function for muscular disabilities related to knee motion like standing up and sitting down. Even though the participants in the experiment were not elderly, this equipment can also be used for seniors, especially those with muscle problems. On the other hand, Lee et al. (2017) developed the lower limb exoskeleton using the EEG signal, with the intention of offering more functionality than a traditional wheelchair. They used healthy participants as a proof of concept for their work, with the target users being people with limited or no residual motor control. This exoskeleton can also be used for elderly people with motor difficulties. The result showed all the subjects successfully performed the main task for the three different directions: walk front, turn left and turn right.

State of the art smart home technologies for elderly people using BCI have been proposed by Jafri et al. (2019) and Chai et al. (2020). Both of the experiments of Jafri and Chai were conducted to test the feasibility of the smart home designs. Although the result of attention level for the younger male reached attention level 74.78 within 26.20 s which was quicker than younger female and older people, the added value for this experiment is the elderly people still can control the function in a smart home using several BCI experimental paradigms (Jafri et al., 2019). One of the smart home services is dialing the three emergency numbers specially designed for older people in hands-free mode using EMG and SSVEP (Chai et al., 2020).

Another important unresolved issue involves the challenges in clinical applications of BCIs with older individuals who have swallowing disorders (e.g., ALS patients with progressive dysphagia), or spinal cord injury [e.g., after spinal shock ends spastic activity may develop in the detrusor muscle restricting the bladder capacity to store urine and resulting in incontinence (Rupp, 2014)]. Moreover, invasive BCIs that require implantation of the device might be a serious ethical issue. Therefore, non-invasive EEG-based BCIs and hBCIs appear to be the most promising technologies. However, hBCI has shown advantages in various applications as it combines the strengths of different BCI paradigms (e.g., high accuracy, minimal daily setup, rapid response times, and multi-functionality). hBCI could further help improve the quality of life of older adults and elderly patients through the development of multifunctional and multidimensional interfaces. Auditory, visual or tactile BCIs may not able to satisfy the requirements of real-life activities, and may not be suitable in terms of comfort for all elderly people due to the potential deficits in hearing, vision, and sensation associated with aging. For example, visual stimuli using P300-based BCI, or SSVEP-based BCI may cause eye fatigue (e.g., imminent retinal fatigue) due to the prolonged visual fixation, or may even harm elderly people who cannot control their gaze (unattainable volitional movements), or have weak vision. These stimuli may also induce epileptic seizures in some patients. In addition, aging affects the integration of temporal rate of auditory flutter (amplitude modulation) presented with visual flicker (Brooks et al., 2015). These age-related changes in auditory and visual interactions in temporal rate perception may affect P300-based BCI performances. However, adding additional non-brain signals or combining more than two BCI modalities may compensate the age-related changes. BCI based on controlling a smart home or an autonomous wheelchair requires multiple degrees of freedom and fast intention detection, making solely EEG-based multiple devices or wheelchair control a challenge. hBCIs may offer more effective control for elderly people, especially by offering multiple commands and accurate stop in emergency cases. In addition, direct tactile stimulation may improve short-term and long-term memory in elderly patients diagnosed with Alzheimer’s disease. These improvements may lead to improved psychological well-being, and increased socialization and participation in daily activities (Witucki and Twibell, 1997; Herweg et al., 2016).

The rapid growth in neuroinformatics and related intelligent algorithms may also advance EEG analyses and help to improve the performance of existing BCIs for the usage at home by reducing the time for the calibration phase and increasing classification accuracy and ITR. With this goal in mind, researchers have been using some common methods for reducing the number of EEG channels, removing artifacts using online source separation, distinguishing between neural activation patterns using machine-learning algorithms, and understanding brain mechanisms using advanced brain network analyses. For example, Sparse Bayesian Learning (Zhang et al., 2015) has been used to predict subject’s behaviors or cognitive states from his brain activities with a small number of samples of high dimensional data (Sparse estimation toolbox: https://bicr.atr.jp/cbi/sparse_estimation/index.html). Deep learning algorithms (Tabar and Halici, 2016; Schwemmer et al., 2018) have also been used to extract useful feature representations from raw data and achieve a high EEG classification accuracy. In addition, the Granger causality methods have been used to assess brain connectivity (Chen et al., 2019).

Developing EEG-based game control may also help elderly people to improve their capacity of multitasking to carry out the tasks in everyday life. This capacity can facilitate the control of smart home appliances, drone swarms, and/or assistive robots. Neurofeedback (biofeedback for the brain) can be an additional option to enhance cognitive performance of elderly people (Jirayucharoensak et al., 2019). The last challenge is the development of hardware and software solutions for home-based applications that can be used by healthy older adults and elderly patients with minimal technical oversight, although there are already many user-friendly, wearable, portable, and wireless EEG equipment in the market such as recoveriX, mindBEAGLE, Unicorn Speller (g.tec medical engineering, Graz, Austria).

## Conclusion

This mini-review has presented several potential BCI applications (e.g., cognitive and motor prosthetics) to assist the older adults and elderly patients using non-invasive measurement. A variety of external aids and neurofeedback tests are available, and have been shown to be useful to and desired by older people, healthcare persons, caretakers and family members. Interactive gaming tests can monitor and improve the cognitive ability of aged people. The current wheelchair and exoskeleton technologies have been developed to support elder people and allow them to carry on their daily routines and at the same time, to provide rehabilitation of deteriorated muscle and motor function. Smart home environments can assist the elderly in living independently and feeling safe in their own homes. We hope that the technologies reviewed in this article will further stimulate the design of new technologies and devices based on BCI for senior citizens. BCI technology has already shown promising results in providing assistance in both cognitive and physical support and rehabilitation, and we look forward to future innovation in this important area of research that affects all of us eventually.

## Author Contributions

All authors were involved in the writing and editing of the manuscript, specific author contributions were: AB overall conceptual design for review, supervisor of NJ – advising in writing and figure design. AB, NJ, and SO contributed to selecting the articles, analyzing results, writing, and editing of the manuscript. JP and CC contributed to writing and editing of the manuscript.

## Conflict of Interest

The authors declare that the research was conducted in the absence of any commercial or financial relationships that could be construed as a potential conflict of interest.

## Footnotes

1 https://www.bci2000.org/

2 https://sccn.ucsd.edu/eeglab/

3 http://www.fieldtriptoolbox.org/

4 http://neuroimage.usc.edu/brainstorm
